# Dental caries and associated factors among diabetic and nondiabetic adult patients attending Bichena Primary Hospital’s Outpatient Department

**DOI:** 10.3389/froh.2022.938405

**Published:** 2022-11-02

**Authors:** Anley Shiferaw, Girma Alem, Mekonnen Tsehay, Getiye Dejenu Kibret

**Affiliations:** ^1^ Department of Public Health, College of Health Sciences, Debre Markos University, Debre Markos, Ethiopia; ^2^ Department of Nursing, College of Health Sciences, Debre Markos University, Debre Markos, Ethiopia; ^3^ Department of Psychiatry, College of Medicine and Health Sciences, Wollo University, Dessie, Ethiopia

**Keywords:** dental caries, diabetics, nondiabetics, patients, Ethiopia

## Abstract

**Purpose:**

Dental caries is a significant public health issue affecting both the diabetic and nondiabetic populations. However, the problem and associated factors of dental caries among diabetics and nondiabetics patients are not well-known in Ethiopia. This study aims to compare the prevalence of dental caries and associated factors among diabetic and nondiabetic patients at the Outpatient Department of Bichena Primary Hospital in Northwest, Ethiopia.

**Methods:**

Institutional based, comparative cross-sectional study was conducted from October 7 to December 6, 2019, among 200 diabetes and 400 nondiabetic adult patients. A consecutive sampling technique was implemented to recruit study participants. Data were collected by a pretested structured questionnaire and analysis was performed in Statistical Package for Social Science version 20. Bivariable and multivariable logistic regressions were employed and variables with a *p*-value < 0.05 were declared statistically significant.

**Results:**

A total of 582 patients were involved in the study with a response rate of 97.0%. The prevalence of dental caries was 67.9% [95% confidence interval (CI): 63.2%–72.8%] and 79.6% (95% CI: 74.0%–85.70%) in nondiabetic and diabetic group, respectively. Females gender [adjusted odds ratio (AOR) = 1.79, 95% CI: 1.15–2.77], poor oral hygiene (AOR = 2.95, 95% CI: 1.71–5.11), lack of regular teeth cleaning habits (AOR = 3.26, 95% CI: 2.13–4.97), feeling dry mouth (AOR = 2.31, 95% CI: 1.11–4.81), sugared tea drinking (AOR = 2.00, 95% CI: 1.18–3.38), inadequate oral health knowledge (AOR = 3.51, 95% CI: 2.19–5.62), and khat chewing (AOR = 2.14, 95% CI: 1.24–3.71) were significantly associated factors with high prevalence of dental caries.

**Conclusion:**

The prevalence of caries was significantly higher among diabetics than nondiabetics. Oral health education with preventive measures such as improving teeth cleaning practice, reducing sugary foods and drinks intake, and improving oral hygiene practice of patients should be mainstreamed along with diabetic follow-up care.

## Introduction

### Background

Dental caries is the localized destruction of susceptible dental hard tissues by acidic by-products from the bacterial fermentation of dietary carbohydrates (1). Dental caries is the most common chronic illness in the world (2, 3), which affects almost half of the world's population (44%) (4, 5). It is also the most prevalent oral condition, with 2.3 billion individuals affected, according to the 2015 Global Burdens of Disease Study (6).

People are at risk of dental caries throughout their lifetimes (1, 5); in particular, diabetic people are at a higher risk of developing caries because of hypo salivation and high salivary glucose levels (7). There is a high correlation between high blood glucose levels and high salivary glucose levels in diabetic patients (8).

Low-income countries have the highest dental caries burden, most of which go untreated (5, 9). The consequences of untreated dental caries can be pain, chewing problems, broken teeth, tooth abscesses, infection, and tooth loss. All of these consequences may lead to recurrent antibiotic prescriptions, distress, and sleep disturbance (10, 11). It also affects eating, learning, communication skills, work performance, recreational activities, growth, and development, and has a long-term psychological effect on the affected patients (12, 13).

Dental caries is becoming more prevalent in Africa (14), with rates reaching 86.63% in Egypt (15), 83.7% in Uganda (16), and 67.9% in Eritrea (17). In Ethiopia, the prevalence of dental caries among adults ranged from 31.5% to 78.2% (18, 19). There is also a difference across Ethiopia, with the uppermost prevalence reported in the Tigray region (46.59%) and the bottom prevalence reported in Addis Ababa (34.20%). The overall prevalence of dental caries in Ethiopia was reported to be 40.98% (20). Dental caries prevalence among diabetic patients has been reported to be 78.9% in India (21), 84.49% in Pakistan (22), and 67% in China (23).

Previous studies reported that age, sex, body mass index, educational level, socioeconomic status, poor oral hygiene, poor tooth cleaning habits, and increasing consumption of sugary food have an impact on dental caries experience (15, 24–26), whereas Diabetic patients, possible risk factors include age, sex, socioeconomic status, feeling of dry mouth during meals (xerostomia), poor dietary control, and inadequate knowledge about their increased risk for oral health illness (22, 27).

Dental caries is a preventable disease (28). World Health Organization (WHO) recommends eating a healthy diet, attending regular dental check-ups, cleaning teeth twice a day with fluoride toothpaste, and avoiding sweetened foods and drinks for dental caries prevention (2, 4, 5).

Dental caries prevalence continues to increase in the African region due to growing consumption of free sugars and inadequate exposure to fluoride (2). Similarly, a study conducted in Ethiopia reported a 78.2% prevalence of dental caries among adults (19). Although research studies in several countries show a high prevalence of dental caries in diabetic patients, there is no research on dental caries in diabetic patients in Ethiopia. In addition, studies conducted in other areas of the country had study populations from dental clinics only. Further, the study includes factors like socioeconomic status using wealth index, oral hygiene status using oral hygiene index-simplified, and body mass index, which are keys for dental caries development. These risk factors are not properly measured using their indexes in previous studies. Therefore, this study aimed to assess the prevalence of dental caries and its associated factors among diabetic patients and nondiabetic patients in Bichena Primary Hospital, 2019.

## Methods and materials

### Study design, study area, and period

An Institutional based comparative cross-sectional study was conducted from October 7, 2019, to December 6, 2019. This study was conducted at Bichena primary hospital outpatient department, located about 265 km away from Addis Ababa, in Bichena town, East Gojjam, Amhara, Ethiopia. It has been functional since April 2015. The catchment population of the hospital reaches 450,000 people. The hospital provides different inpatient and outpatient services including dental health services and follow-up health services to the population in the surrounding area of Enemay woreda and the nearby districts including Enarj Eawga woreda, Debay Tilat Gin woreda, and Shebel Berenta Woreda.

### Source population

All diabetic and nondiabetic adult patients, who were utilizing health services at Bichena Primary Hospital outpatient department in the last 1 year.

### Study population

For diabetic patients: Those diabetic patients who were available during the data collection time during routine working hours.

For nondiabetics: Those patients who were available during the data collection time during working hours in the outpatient department.

#### Inclusion criteria

Age greater than or equal to 18 years old.

Known diabetic patients.

Those individuals with random blood sugar levels less than 200 mg/dl or fasting blood sugar less than 126 mg/dl for nondiabetic patients.

#### Exclusion criteria

Those who are unable to respond to interviews like acute psychosis patients.

Those who have any physical disorders not permitting oral examination like temporomandibular joint disorders.

Individuals suffering from systemic illness, pregnant mothers.

Unwillingness to participate.

### Study variables

The outcome variable was dental caries among diabetic and nondiabetic adult patients. On the contrary, the explanatory variables were age, sex, body mass index, religion, educational status, marital status, occupation, residence, wealth index, oral hygiene status, tooth brushing habit, feeling dry mouth, history of dental visits, alcohol consumption, sugared foods/drinks, cigarette smoking, and khat chewing.

### Operational definitions

**Dental caries:** the presence of tooth decay, or missing or filled teeth at the time of the oral examination.

**Sound tooth:** a tooth is recorded as sound if it shows no evidence of treated or untreated clinical caries.

**Clinical caries:** defined as a cavity diagnosed by visual examination/probing of the mouth.

**Filled tooth due to caries:** a tooth is considered filled when it has one or more permanent restorations due to the presence and/or history of tooth decay.

**Missed tooth due to caries:** a tooth is considered missed when it has been permanently extracted because of caries. Excluding permanent teeth missing due to any other reason like absent congenitally, extracted for orthodontic reasons or because of periodontal disease, trauma.

**Decayed, missing and filled teeth (DMFT)-index per person:** the average number of permanent teeth per person that are decayed (D), Missing (M), and Filled (F) teeth because of caries.

**Diabetes mellitus:** diagnosed in participants with fasting blood glucose levels greater than or equal to 126 mg/dl (7.0 mmol/L), or with normal glucose but under treatment (diet or medical) for diabetes. Fasting is defined as no caloric intake for at least 8 h.

**Nondiabetic patients:** diagnosed in participants with fasting blood sugar levels less than 126 mg/dl or random blood sugar less than 200 mg/dl and who are not with diet or medical treatment.

**Simplified oral hygiene index (OHI-S):** a method by which the extent of debris (soft foreign material loosely attached to the tooth) and the extent of calculus (hardening foreign material firmly attached to the tooth) on six surfaces of six preselected teeth is estimated (29).

**Oral hygiene status:** oral hygiene will be classified into good, fair, and poor using the OHI-S scores as follows: Good (0–1.2 OHI-S score), Fair (1.3–3.0 OHI-S score), and Poor (3.1–6.0 OHI-S score) (29).

**Adequate knowledge:** respondents were considered to be knowledgeable if he/she is correctly answered greater than five of the total knowledge assessing questions (30).

**Inadequate knowledge:** respondents were considered inadequately knowledgeable if he/she was correctly answered less than or equal to five of the total knowledge assessing questions.

### Sample size determination and sampling technique

#### Sample size determination

The sample size of the study was determined by using factors significantly associated with dental caries by considering the following assumptions: two-sided confidence level (95%), power 80%, the ratio of exposures to no exposures 1:2, considering experiencing tooth pain as significant determinants of dental caries and the proportion in the exposed group was 75.3%, the proportion in the unexposed group was 62.8% with an adjusted odds ratio (AOR) of 1.78 (31). Using EPI-info version 6, StatCalc toolbar auto-calculator software the calculated sample size was 600 adult patients (200 for diabetes and 400 for nondiabetic patients) including a 10% nonresponse rate. A consecutive sampling technique was used to select study participants until the required sample size was reached.

### Data collection tools and procedures

Data were collected using a pretested structured interviewer-administered questionnaire and oral examination. The questionnaire was developed from WHO oral health survey with modifications (32). The questionnaire was prepared in English, translated into the local language Amharic, and back into English to check its consistency. Oral examination was performed by trained nurses for dental caries and oral hygiene status. The agreement of the examiners in the detection of dental caries was analyzed using Cohen's kappa statistics for inter-and intraexaminer reliability giving values ranging from 0.87 to 0.96 and 0.96 to 1.0, respectively.

Dental caries was evaluated using the WHO oral health assessment form for adults by assessing DMFT index (32). Oral hygiene status was also assessed using the oral hygiene index-simplified by examination of dental debris and calculus on specific preselected surfaces of teeth (29). According to their time of feeding, RBS (random blood sugar) or FBS (fasting blood sugar) was determined by the data collector.

Body weight was measured using a digital weighing scale (Adult Scale ASTOR) to the nearest 0.1 kg with the participants barefooted and wearing light clothes. Height was measured using Adult Scale ASTOR to the nearest 0.1 cm with the shoes and any hats of the study participant removed. The weighing scales were checked and adjusted at zero level between each measurement and were tested for repeatability of the measures. Participants were standing upright with the head, shoulder, buttock, lower limb, and heel of the foot touching the vertical stand. Body mass index was calculated as weight in kilograms over height in meters squared and was categorized as less than 18.50 (below normal weight), 18.50–24.99 (normal weight), greater than or equal to 25.00 (overweight).

The household wealth index (socioeconomic status) was assessed based on ownership of selected household items. These variables were collected using Ethiopian Demographic and Health Survey 2016 household characteristics questionnaire. The items include: Electricity, Radio, Television, Computer, Refrigerator, Chair, Bed with cotton/spring mattress, Electric mitad, Mobile telephone, a means of transportation, Bicycle, Motorcycle, Animal-drawn cart, Car or Truck, Bajaj, What is your monthly Income, Bank account, Owns a house, agricultural land, Number of members per sleeping room, Cows/bulls, Horses/donkeys/mules, Goats/Sheep, Chickens, Beehives, Source of drinking water, Type of toilet facility, Type of cooking fuel, and Type of flooring.

The data quality was maintained through careful design and pretesting of the questionnaire. Two days of training were given to data collectors on the objectives of the study, how to interview, how to collect data from patients, and maintain confidentiality. Then after providing the training, a pretest was conducted on 5% of the sample size prior to the actual data collection period at Debrework primary hospital. The principal investigator was closely monitoring the data collection process to ensure the completeness of questions. In addition to the above, data were rechecked during data entry before analysis, to prevent missing important data. Incomplete questionnaires were discarded from the analysis.

### Data processing and analysis

The collected data were cleared, coded, and entered in Epidata version 3.1 and exported to Statistical Package for Social Science (SPSS) version 20.0 (IBM SPSS Statistics for Windows, version 20 IBM Corp., Armonk, NY, USA)) for further analysis. Descriptive (frequency distribution tables, mean and standard deviation), as well as inferential analysis, was performed. Categorization was done for continuous variables using information from different pieces of literature. Cohen's kappa coefficients were used to assess inter- and intraexaminer agreements. Principal component analysis was done to construct the household wealth index by asking for all assets they have. Communality value >0.5, Kaiser–Meyer–Olkin (KMO) (sampling adequacy) with 0.87, and complex structure factor (eigenvalue) greater than 1 were considered.

Bivariate and multivariable were done to identify significantly associated variables with the dependent variable. Model fitness was checked using the Hosmer–Lemeshow test with (*p*-value < 0.05). A backward stepwise logistic regression model was used during multivariable logistic regression to control confounding effects. From the bivariate analysis, variables with *p* < 0.25 were considered for multivariable analysis. From the multivariable logistic regression analysis, variables with a significance level of *p* < 0.05 were taken as statistically significant and independently associated with dental caries.

#### Ethical considerations

A letter of ethical approval was obtained from the Ethical Review Board (ERB) of Debre Markos University, College of Health Sciences (HSC/R/C/Ser/Co/253/11/12). Furthermore, before the data collection, a formal permission letter was obtained from Bichena Primary Hospital to the outpatient department. In addition, written informed consent was obtained from respondents to confirm their willingness to participate in the study after explaining the objective of the study. The respondents noticed that they had the right to refuse or terminate at any point during the interview. The information provided by each respondent was kept confidential. Those identified as having dental caries and fasting blood sugar greater than 125 mg/dl were referred to the dental and diabetics outpatient department for better treatment and investigation.

## Results

### Sociodemographic and clinical characteristics

Five hundred eighty-two patients (386 nondiabetics and 196 diabetics) participated (with a response rate of 97.0%). The age distribution of the study participants in the diabetic group was with normal age group pattern (18- to 70-year old) and the mean age of the group was 40.5 (±13.7) years. The nondiabetic group was with normal age group pattern (20–75 years old) and the mean age of the group was 47.3 (±12.7) years (Table 1).

**Table 1:** Sociodemographic and clinical characteristics of study participants attending the outpatient department in Bichena Primary Hospital (BPH) in northwest Ethiopia, 2019 (*n* = 582).

Variables	Nondiabetics (*n* = 386)	Diabetics (*n* = 196)	Total	Chi-square
	Number (%)	Number (%)	Number (%)	*p*-value
Dental caries				
No	124 (32.1)	40 (40.4)	164 (28.2)	0.003
Yes	262 (67.9)	156 (79.6)	418 (71.8)	
Decayed teeth (mean ± SD)	1.52 (±1.46)	1.51 (±1.60)	1.52 (±1.51)	
Missed teeth (mean ± SD)	0.23 (±0.58)	0.11 (±0.33)	0.18 (±0.51)	
Filled teeth (mean ± SD)	0.01 (±0.13)	0.05 (±0.21)	0.02 (±0.16)	
DMFT index (mean ± SD)	1.75 (±1.78)	1.66 (±1.79)	1.72 (±1.78)	
Age				
18–29	103 (26.7)^a^	20 (10.2)	123 (21.1)	<0.001
30–39	99 (25.7)	40 (20.4)	139 (23.9)	
40–49	75 (19.4)	52 (26.5)^b^	127 (21.8)	
50–59	71 (18.4)	49 (25.0)^b^	120 (20.6)	
≥60	38 (9.8)	35 (17.9)^b^	73 (12.5)	
Sex				
Male	178 (46.1)	115 (58.7)	293 (50.3)	0.005
Female	208 (53.9)	81 (41.3)	289 (49.7)	
BMI				
<Normal weight	12 (3.1)	6 (3.1)	18 (3.1)	<0.001
Normal weight	300 (77.7)^a^	106 (54.1)	406 (69.8)	
Overweight	74 (19.2)	84 (42.9)^b^	158 (27.1)	
Residence				
Urban	239 (61.9)	105 (53.6)	344 (59.1)	0.065
Rural	147 (38.1)	91 (46.4)	238 (40.9)	
Religion				
Orthodox	243 (63.0)^a^	96 (49.0)	339 (58.2)	0.003
Muslim	108 (28.0)	75 (38.3)^b^	183 (31.4)	
Others	35 (9.0)	25 (12.8)	60 (10.3)	
Marital status				
Single	76 (19.7)^a^	17 (8.7)	93 (16.0)	0.002
Married	286 (74.1)	163 (83.2)^b^	449 (77.1)	
Widowed/divorced	24 (6.2)	16 (8.2)^b^	40 (6.9)	
Level of education				
Illiterate	98 (25.4)	75 (38.3)^b^	173 (29.7)	<0.001
Read and write	82 (21.2)	59 (30.1)^b^	141 (24.2)	
Undergraduate	102 (26.4)^a^	37 (18.9)	139 (23.9)	
Graduate	104 (26.9)^a^	25 (12.8)	129 (22.2)	
Occupation				
Farmer	122 (31.6)	61 (31.1)	183 (31.4)	0.063
Gov't employed	122 (31.6)^a^	34 (17.3)	156 (26.8)	
Merchant	90 (23.3)	71 (36.2)^b^	161 (27.7)	
Others	52 (13.5)	30 (15.3)	82 (14.1)	
Wealth index				
Lower class	126 (33.0)	65 (33.2)	191 (33.0)	0.473
Middle class	136 (35.6)	59 (30.1)	195 (33.7)	
Upper class	120 (31.4)	72 (36.7)	192 (33.3)	

^a^ Using Kruskal–Wallis test pairwise comparison: nondiabetic variable subgroups that show a difference.

^b^ Using Kruskal–Wallis test pairwise comparison: diabetic variable subgroups that show a difference.

SD, standard deviation; BMI, body mass index.

### Lifestyle characteristics and food consumption patterns

This study showed that 72 (18.7%) of nondiabetics and 85 (43.4%) of diabetics were khat chewers. Similarly, 15 (3.88%) of diabetics and 10 (5.10%) of nondiabetics were cigarette smokers (Table 2).

**Table 2:** Lifestyle characteristics and food consumption patterns of study participants attending the outpatient department in BPH, northwest Ethiopia, 2019.

Variables	Nondiabetic	Diabetic	Total
	Number (%)	Number (%)	Number (%)
Dental caries			
No	124 (32.1)	40 (40.4)	164 (28.2)
Yes	262 (67.9)	156 (79.6)	418 (71.8)
Decayed teeth (mean ± SD)	1.52 (±1.46)	1.51 (±1.60)	1.52 (±1.51)
Missed teeth (mean ± SD)	0.23 (±0.58)	0.11 (±0.33)	0.18 (±0.51)
Filled teeth (mean ± SD)	0.01 (±0.13)	0.05 (±0.21)	0.02 (±0.16)
DMFT index (mean ± SD)	1.75 (±1.78)	1.66 (±1.79)	1.72 (±1.78)
Usually eat for breakfast			
Bread/pasta	170 (44.0)	14 (7.1)	184 (31.6)
Injera (wot or firfir)	216 (56.0)	182 (92.9)	398 (68.4)
Usually eat for lunch			
Bread/pasta	30 (7.8)	12 (6.1)	42 (7.2)
Injera (wot or firfir)	356 (92.2)	184 (93.9)	540 (92.8)
Usually eat for dinner			
Bread/pasta	18 (4.7)	7 (3.6)	25 (4.3)
Injera (wot or firfir)	368 (95.3)	189 (96.4)	557 (95.7)
Drink sugared tea			
No	61 (15.8)	56 (28.6)	117 (20.1)
Yes	325 (84.2)	140 (71.4)	465 (79.9)
Drink sugared coffee			
No	112 (29.0)	88 (44.9)	200 (34.4)
Yes	274 (71.0)	108 (55.1)	382 (65.6)
Soft drinks			
No	157 (40.7)	115 (58.7)	272 (46.7)
Yes	229 (59.3)	81 (41.3)	310 (53.3)
Soft food staff			
No	211 (54.7)	155 (79.1)	366 (62.9)
Yes	175 (45.3)	41 (20.9)	216 (37.1)
Alcohol drinking			
No	135 (35.0)	116 (59.2)	251 (43.1)
Yes	251 (65.0)	100 (40.8)	331 (56.9)
Khat chewing			
No	314 (81.3)	111 (56.6)	425 (73.0)
Yes	72 (18.7)	85 (43.4)	157 (27.0)
Cigarette smoking			
No	371 (96.11)	186 (94.90)	557 (95.71)
Yes	15 (3.88)	10 (5.10)	25 (4.29)

BPH, Bichena Primary Hospital; SD, standard deviation.

### Knowledge and hygiene practice on oral health

Assessment of the responses to the ten questions related to oral health knowledge showed that 245 (63.50%) nondiabetics and 88 (44.90%) diabetics had adequate knowledge about dental caries. Additionally, 180 (46.60%) nondiabetics and 56 (28.90%) diabetic patients reported that they had teeth cleaning practices (Table 3).

**Table 3:** Knowledge and practice on oral health of study participants attending the outpatient department in BPH, northwest Ethiopia, 2019.

Variables	Nondiabetics	Diabetics	Total
	Number (%)	Number (%)	Number (%)
Knowledge			
Inadequate	141 (36.5)	108 (55.1)	249 (42.8)
Adequate	245 (63.5)	88 (44.9)	333 (57.2)
Dental caries			
No	124 (32.1)	40 (40.4)	164 (28.2)
Yes	262 (67.9)	156 (79.6)	418 (71.8)
Decayed teeth (mean ± SD)	1.52 (±1.46)	1.51 (±1.60)	1.52 (±1.51)
Missed teeth (mean ± SD)	0.23 (±0.58)	0.11 (±0.33)	0.18 (±0.51)
Filled teeth (mean ± SD)	0.01 (±0.13)	0.05 (±0.21)	0.02 (±0.16)
DMFT index (mean ± SD)	1.75 (±1.78)	1.66 (±1.79)	1.72 (±1.78)
Oral hygiene status*			
Good	108 (28.0)	44 (22.5)	152 (26.1)
Fair	163 (42.2)	69 (35.2)	232 (39.9)
Poor	115 (29.8)	83 (42.3)	198 (34.0)
Tooth cleaning habit			
No	206 (53.4)	140 (71.4)	346 (59.5)
Yes	180 (46.6)	56 (28.9)	236 (40.5)
Frequency of cleaning?			
Sometimes	74 (41.1)	20 (35.7)	94 (39.8)
Once per day	67 (37.2)	22 (39.3)	89 (37.7)
Twice per day	39 (21.7)	14 (25.0)	53 (22.5)
Use toothpaste			
No	80 (44.4)	28 (50.0)	108 (45.8)
Yes	100 (55.6)	28 (50.0)	128 (54.2)
Time of cleaning?			
Before/after meals	50 (27.8)	8 (14.3)	58 (24.6)
Before and after meals	71 (39.4)	30 (53.6)	101 (42.8)
At my convenience	59 (32.8)	18 (32.1)	77 (32.6)
No-minutes			
For <2 min	64 (35.6)	20 (35.7)	84 (35.6)
For ≥2 min	116 (64.4)	36 (63.4)	152 (64.4)
Use to clean teeth			
Mefakia	97 (53.9)	29 (51.8)	126 (53.4)
Toothbrush	83 (46.1)	27 (48.2)	110 (46.6)
Reason for cleaning			
To prevent decay	59 (32.8)	9 (16.1)	68 (28.8)
To feel healthy	121 (67.2)	47 (83.9)	168 (71.2)
Reasons for not cleaning			
No use	36 (17.5)	19 (13.6)	55 (15.9)
I don't know the benefit	49 (23.8)	51 (36.4)	100 (28.9)
Negligence	121 (58.7)	70 (50.0)	191 (55.2)
Dry mouth			
No	358 (92.7)	128 (65.3)	486 (83.5)
Yes	28 (7.3)	68 (34.7)	96 (16.5)
Toothache			
No	196 (50.8)	60 (30.6)	256 (44.0)
Yes	190 (49.2)	136 (69.4)	326 (56.0)
Treatment			
Health institution	157 (82.6)	128 (94.1)	285 (87.4)
Traditional healer	33 (17.4)	8 (5.9)	41 (12.6)
Visited a dentist before			
No	250 (67.4)	110 (56.1)	370 (63.6)
Yes	126 (32.6)	86 (43.9)	212 (36.4)
Reason			
Tooth extraction	49 (38.9)	39 (45.3)	88 (41.5)
Tooth filling	4 (3.2)	0	4 (1.9)
Tooth cleaning	33 (26.2)	11 (12.8)	44 (20.8)
Reliving pain	40 (31.7)	36 (41.9)	76 (35.8)
When you have to visit dentist			
Regularly (6–12 months)	32 (8.3)	22 (11.2)	54 (9.3)
Occasionally	53 (13.7)	40 (20.4)	93 (16.0)
Only with dental pain	208 (53.9)	91 (46.4)	299 (51.4)
I don't know	93 (24.1)	43 (21.9)	136 (23.4)

BPH, Bichena Primary Hospital; SD, standard deviation.

*Using Kruskal–Wallis test pairwise comparison, poor oral hygiene status of diabetics shows a difference with good oral hygiene status (*p*-value < 0.033) and fair oral hygiene status (*p*-value < 0.023) of nondiabetics.

The fair oral hygiene status of study participants covers 42.20% of nondiabetics. Poor oral hygiene status was reported in 115 (29.8%) nondiabetics. The oral hygiene status of diabetic patients shows that 69 (35.20%) and 83 (42.3%) of study participants had fair and poor oral hygiene status respectively (Table 3).

### Prevalence of dental caries

**Figure 1:**
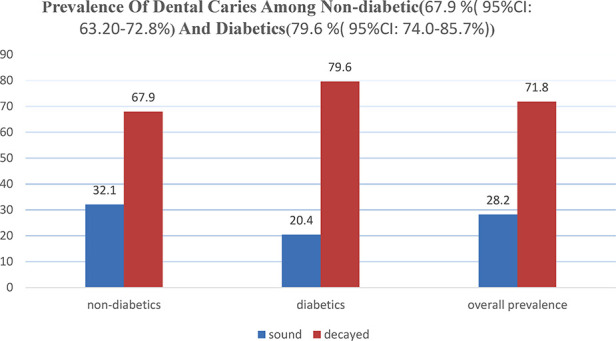
Prevalence of dental caries among nondiabetic and diabetic patients attending the outpatient department in BPH, northwest Ethiopia, 2019. BPH, Bichena Primary Hospital.

Of the 582 study participants' teeth examined, 418 (71.8%) were found to have at least one tooth decayed, missed, or filled due to dental caries. The prevalence of dental caries in nondiabetic group was 67.9 [95% confidence interval (CI): 63.2%–72.8%] and for diabetics 79.6% (95% CI: 74.0%–85.70%). The prevalence of dental caries in nondiabetics and diabetics shows a significant difference (chi-square = 8.81: *p*-value = 0.003) (Figure 1).

### Determinants of dental caries

#### Factors associated with dental caries among nondiabetics

During binary logistic regression analysis; sex, body mass index, level of education, simplified oral hygiene index, teeth cleaning habit, frequency of dental visit, feeling of dry mouth during meals, oral health-related knowledge, sugared tea drinking, sugared coffee drinking, alcohol drinking and khat chewing candidate (*p*-value < 0.25) for multiple logistic regression among non-diabetes.

Whereas, the multiple logistic regressions revealed that sex (AOR = 1.98, 95% CI: 1.21–3.24), poor oral hygiene (AOR = 2.75, 95% CI: 1.41–5.36), lack of regular teeth cleaning habits (AOR = 3.86, 95% CI: 2.35–6.34), inadequate oral health-related knowledge (AOR = 3.31, 95% CI: 1.90–5.76), and khat chewing (AOR = 2.25, 95% CI: 1.11–4.57) were significantly associated with dental caries among nondiabetic (Table 4).

**Table 4:** Associated factors of dental caries among nondiabetics from study participants attending the outpatient department in Bichena Primary Hospital in Northwest Ethiopia, 2019.

Variables	Dental caries among nondiabetics				
	No	Yes	COR (95% CI)	AOR (95% CI)	*p*-value
	Number (%)	Number (%)			
Sex					
Male	69 (38.8)	109 (61.2)	1	1	
Female	55 (26.4)	153 (73.6)	1.76 (1.14–2.71)	1.98 (1.21–3.24)	0.006*
Residence					
Urban	84 (35.1)	155 (64.9)	1	1	
Rural	40 (27.2)	107 (72.8)	1.45 (0.92–2.27)	1.46 (0.83–2.57)	0.183
Level of education					
Graduate	40 (38.5)	64 (61.5)	1	1	
No formal education	24 (24.5)	74 (75.5)	1.92 (1.05–3.53)	0.70 (0.33–1.49)	0.362
Read and write	25 (30.5)	57 (69.5)	1.42 (0.77–2.63)	1.10 (0.52–2.32)	0.785
Undergraduate	35 (34.3)	67 (65.7)	1.19 (0.67–2.11)	0.72 (0.36–1.41)	0.347
BMI					
Underweight	6 (50.0)	6 (50.0)	1	1	
Normal weight	99 (33.0)	201 (67.0)	2.03 (0.63–6.45)	1.30 (0.35–4.79)	0.694
Overweight	19 (25.7)	55 (74.3)	2.89 (0.83–10.06)	1.79 (0.44–7.23)	0.412
Teeth cleaning habit					
No	36 (17.5)	170 (82.5)	4.51 (2.84–7.17)	3.86 (2.35–6.34)	0.000*
Yes	88 (48.9)	92 (51.1)	1	1	
Dry mouth					
No	119 (33.2)	239 (66.8)	1	1	
Yes	5 (17.9)	23 (82.1)	2.29 (0.85–6.17)	2.72 (0.94–7.83)	0.063
How often have you visited the dental clinic					
Regularly	15 (46.9)	17 (53.1)	1	1	
Occasionally	19 (35.8)	34 (64.2)	1.57 (0.64–3.85)	1.21 (0.42–3.50)	0.715
With dental pain	59 (28.4)	149 (71.6)	2.22 (1.04–4.75)	1.50 (0.62–3.62)	0.363
I don't know	31 (33.3)	62 (66.7)	1.76 (0.77–3.99)	0.93 (0.35–2.46)	0.898
Sugared tea drinking					
No	27 (44.3)	34 (55.7)	1	1	
Yes	97 (29.8)	228 (70.2)	1.86 (1.06–3.26)	1.67 (0.84–3.32)	0.142
Coffee drinking					
No	45 (40.2)	67 (59.8)	1	1	
Yes	79 (28.8)	195 (71.2)	1.65 (1.04–2.62)	1.01 (0.55–1.86)	0.960
Alcohol drinking					
No	49 (36.3)	86 (63.7)	1	1	
Yes	75 (29.9)	176 (70.1)	1.33 (0.85–2.08)	1.47 (0.88–2.47)	0.141
Khat chewing					
No	111 (35.4)	203 (64.6)	1	1	
Yes	13 (18.1)	59 (81.9)	2.48 (1.30–4.72)	2.25 (1.11–4.57)	0.024*
Oral health knowledge					
Good	25 (17.7)	116 (82.3)	1	1	
Poor	99 (40.4)	146 (59.6)	3.14 (1.90–5.19)	3.31 (1.90–5.76)	0.000*
Oral hygiene index					
Good	43 (39.8)	65 (60.2)	1	1	
Fair	57 (35.0)	106 (65.0)	1.23 (0.74–2.03)	0.92 (0.52–1.64)	0.800
Poor	24 (20.9)	91 (79.1)	2.50 (1.38–4.53)	2.75 (1.41–5.36)	0.003*

AOR, adjusted odds ratio; CI, confidence interval; BMI, body mass index; COR, crude odds ratio.

*indicates significantly associated.

#### Factors associated with dental caries among diabetics

During binary logistic regression analysis, potential dental caries associated factors that showed a *p*-value < 0.25 were; wealth index, level of education, simplified oral hygiene index, teeth cleaning habit, visiting dental clinic before, frequency of dental visit, feeling of dry mouth during meals, toothache, oral health-related knowledge, sugared tea drinking, sugared coffee drinking and khat chewing candidate for multiple logistic regression among diabetes.

Whereas, the multiple logistic regression revealed that fair simplified oral hygiene index (AOR = 3.05, 95% CI: 1.04–8.97), poor oral hygiene (AOR = 5.13, 95% CI: 1.77–14.91), feeling dry mouth during meals (AOR = 3.16, 95% CI: 1.11–8.98), inadequate oral health-related knowledge (AOR = 5.24, 95% CI: 2.12–12.96), visiting dental clinic before (AOR = 2.66, 95% CI: 1.02–6.92), and toothache (AOR = 2.43, 95% CI: 1.01, 5.79) were significantly associated with dental caries among diabetic patients (Table 5).

**Table 5:** Associated factors of dental caries among diabetics from study participants attending the Outpatient Department in Bichena Primary Hospital, northwest Ethiopia, 2019.

Variables	Dental caries among nondiabetics				
	No	Yes	COR (95% CI)	AOR (95% CI)	*p*-value
	Number (%)	Number (%)			
Level of education					
Graduate	9 (36.0)	16 (64.0)	1	1	
No formal education	11 (14.7)	64 (85.3)	3.27 (1.05–3.53)	1.85 (0.49–7.01)	0.360
Read and write	11 (18.6)	48 (81.4)	2.45 (0.86–6.99)	2.34 (0.60–9.01)	0.216
Undergraduate	9 (24.3)	28 (75.7)	1.75 (0.57–5.30)	1.36 (0.32–5.73)	0.675
Wealth index					
Lower class	18 (27.70)	47 (72.30)	1	1	
Middle class	8 (13.60)	51 (86.40)	2.44 (0.97–6.14)	2.35 (0.74–7.45)	0.145
Higher class	14 (19.40)	58 (80.60)	2.14 (0.99–4.62)	1.38 (0.49–3.85)	0.539
Teeth cleaning habit					
No	22 (15.7)	118 (84.3)	2.54 (1.23–5.23)	1.96 (0.79–4.87)	0.144
Yes	18 (32.1)	38 (97.9)	1	1	
Dry mouth					
No	34 (26.6)	94 (73.4)	1	1	
Yes	6 (8.8)	62 (91.2)	3.73 (1.48–9.42)	3.16 (1.11–8.98)	0.031*
Toothache					
No	18 (26.9)	49 (73.1)	1	1	
Yes	22 (17.1)	107 (82.9)	1.78 (0.87–3.62)	2.43 (1.01–5.79)	0.045*
Visit dentist before					
No	30 (27.3)	80 (72.7)	1	1	
Yes	10 (11.6)	76 (88.4)	2.85 (1.30–6.22)	2.66 (1.02–6.92)	0.044*
How often you visited dental clinic					
Regularly	8 (36.4)	14 (63.6)	1	1	
Occasionally	9 (22.5)	31 (77.5)	1.96 (0.62–6.17)	1.40 (0.29–6.69)	0.666
With dental pain	15 (16.5)	76 (83.5)	2.89 (1.03–8.11)	2.15 (0.55–8.40)	0.271
I don't know	8 (18.6)	35 (81.4)	2.50 (0.78–7.97)	1.95 (0.41–9.28)	0.399
Sugared tea drinking					
No	17 (30.4)	39 (69.6)	1	1	
Yes	23 (16.4)	117 (83.6)	2.21 (1.07–4.57)	2.22 (0.93–5.27)	0.070
Sugared coffee drinking					
No	22 (25.0)	66 (75.0)	1	1	
Yes	18 (16.7)	90 (83.3)	1.66 (0.82–3.35)	2.32 (0.96–5.57)	0.059
Khat chewing					
No	29 (26.1)	82 (73.9)	1	1	
Yes	11 (12.9)	74 (87.1)	2.37 (1.11–5.09)	1.84 (0.70–4.86)	0.213
Oral health knowledge					
Poor	10 (9.3)	98 (90.7)	5.06 (2.31–11.12)	5.24 (2.12–12.96)	0.000*
Good	30 (34.1)	58 (65.9)	1	1	
Oral hygiene index					
Good	16 (36.4)	28 (63.6)	1	1	
Fair	11 (15.9)	58 (84.1)	3.01 (1.23–7.34)	3.05 (1.04–8.97)	0.042*
Poor	13 (15.7)	70 (84.3)	3.07 (1.31–7.22)	5.13 (1.77–14.91	0.003*

AOR, adjusted odds ratio; CI, confidence interval; BMI, body mass index; COR, crude odds ratio.

*indicates significantly associated.

#### Factors associated with dental caries among nondiabetics and diabetics

During binary logistic regression analysis; sex, residence, body mass index, level of education, simplified oral hygiene index, teeth cleaning habit, frequency of dental visit, visiting dental clinic before, feeling of dry mouth during meals, oral health-related knowledge, sugared tea drinking, sugared coffee drinking, toothache and khat chewing candidate (*p*-value < 0.25) for multiple logistic regression.

Whereas, the multiple logistic regression revealed that being female (AOR = 1.79, 95% CI: 1.15–2.77), having poor oral hygiene status (AOR = 2.95, 95% CI: 1.71–5.11), not cleaning teeth AOR = 3.26, 95% CI: 2.13–4.97), inadequate oral health-related knowledge (AOR = 3.51, 95% CI: 2.19–5.62), feeling of dry mouth during meals (AOR = 2.31, 95% CI: 1.11–4.81), sugared tea drinking (AOR = 2.00, 95% CI: 1.18–3.38), and khat chewing (AOR = 2.14, 95% CI: 1.24–3.71) were found significantly linked with dental caries (Table 6).

**Table 6:** Associated factors of dental caries among study participants attending the outpatient department in BPH, Northwest Ethiopia, 2019.

Variable	Dental caries				
	Yes	No	COR (95% CI)	AOR (95% CI)	*p*-value
	Number (%)	Number (%)			
Sex					
Female	219 (52.4)	70 (42.7)	1.47 (1.02–2.12)	1.79 (1.15–2.77)	0.009*
Male	199 (47.6)	94 (57.3)	1	1	
Body mass index					
Normal weight	288 (68.9)	118 (72.0)	1.95 (0.75–5.06)	0.83 (0.25–2.69)	0.760
Overweight	120 (28.7)	38 (23.2)	2.52 (0.93–6.85)	0.91 (0.55–1.50)	0.730
<Normal weight	10 (2.4)	8 (4.9)	1	1	
Residence					
Rural	182 (43.5)	56 (34.1)	1.48 (1.02–2.16)	0.63 (0.39–1.00)	0.055
Urban	236 (56.5)	108 (65.9)	1	1	
Education					
No formal education	138 (33.0)	35 (21.3)	2.41 (1.44–4.03)	0.93 (0.49–1.74)	0.829
Read and write	105 (25.1)	36 (22.0)	1.78 (1.06–3.00)	1.26 (0.68–2.32)	0.453
Undergraduate	95 (22.7)	44 (26.8)	1.32 (0.79–2.18)	0.78 (0.43–1.41)	0.426
Graduate	80 (19.1)	49 (29.9)	1	1	
Simplified oral hygiene index					
Fair	164 (39.2)	68 (41.4)	1.53 (0.99–2.35)	1.16 (0.71–1.91)0	0.539
Poor	161 (38.6)	37 (22.6)	2.76 (1.70–4.47)	2.95 (1.71–5.11)	<0.001*
Good	93 (22.2)	59 (36.0)	1	1	
Teeth cleaning habit					
No	288 (68.9)	58 (35.4)	4.04 (2.76–5.92)	3.26 (2.13–4.97)	<0.001*
Yes	130 (31.10)	106 (64.6)	1	1	
Dry mouth					
Yes	85 (20.3)	11 (6.7)	3.55 (1.84–6.84)	2.31 (1.11–4.81)	0.025*
No	333 (79.7)	153 (93.3)	1	1	
Toothache					
Yes	252 (60.3)	74 (45.1)	1.84 (1.28–2.65)	1.31 (0.84–2.06)	0.228
No	166 (39.7)	90 (54.9)	1	1	
Visit dentist before					
Yes	166 (39.7)	46 (28.0)	1.69 (1.14–2.50)	1.54 (0.98–2.43)	0.057
No	252 (60.3)	118 (72.00	1	1	
Frequency of visiting a dentist					
Occasionally	65 (15.6)	28 (17.1)	1.72 (0.85–3.46)	1.20 (0.52–2.76)	0.660
With dental pain	225 (53.8)	74 (45.1)	2.25 (1.23–4.11)	1.56 (0.77–3.15)	0.209
I do not know	97 (23.2)	39 (23.8)	1.84 (0.95–3.55)	1.09 (0.50–2.36)	0.817
Regularly	31 (7.4)	23 (14.0)	1	1	
Oral health-related knowledge					
Inadequate	214 (51.2)	35 (21.3)	3.86 (2.54–5.88)	3.51 (2.19–5.62)	<0.001*
Adequate	204 (48.8)	129 (78.7)	1	1	
Sugared tea drinking					
Yes	345 (82.5)	120 (73.2)	1.73 (1.13–2.65)	2.00 (1.18–3.38)	0.010*
No	73 (17.5)	44 (26.8)	1	1	
Sugared coffee drinking					
Yes	285 (68.2)	97 (59.1)	1.48 (1.01–2.15)	1.45 (0.92–2.30)	0.107
No	133 (31.8)	67 (40.9)	1	1	
Khat chewing					
Yes	133 (31.8)	24 (14.6)	2.72 (1.68–4.39)	2.14 (1.24–3.71)	0.006*
No	285 (68.2)	140 (85.4)	1	1	
Wealth index					
Lower class	54 (28.1)	138 (71.9)	1.11 (0.72–1.73)	1.24 (0.72–2.14)	0.423
Middle class	51 (26.0)	145 (74.0)	1.24 (0.79–1.93)	1.00 (0.58–1.71)	0.995
Higher class	59 (30.4)	135 (69.6)	1	1	

AOR, adjusted odds ratio; CI, confidence interval; BPH, Bichena Primary Hospital; COR, crude odds ratio.

*indicates significantly associated.

## Discussion

In this study, the prevalence of dental caries was 79.6% in diabetics and 67.9% in nondiabetics. This demonstrates that the prevalence of dental caries varies significantly between groups. This statistically significant difference is supported by studies conducted in Chennai, India (27), Jammu and Kashmir in India (7), in Karachi, Pakistan (33). The justification for this may be a higher content of salivary glucose in diabetic patients (8) as well as a lack of awareness among diabetic patients about oral complications. On the other hand, some studies from Uruguay (34) and Pakistan (35) reported no significant difference. The possible reason for these discrepancies might be due to the poor metabolic control of diabetics (36).

In contrast, a study from Tumkur, South India, reported a higher prevalence of dental caries among nondiabetics (37). The possible explanation for this discrepancy may be good awareness of diabetic patients on diet management practices, like sugar-free diets (37). Another reason might be the difference in frequency of visiting a dentist, which facilitates early detection and treatment (38).

The prevalence of dental caries among diabetics (79.6%) was in line with the study reported from Asmara, Eritrea, 79% (39), and from New Delhi, India (78.9%) (21). However, it is less than that of a study done in Pakistan (83.85%) (22). On the other hand, the prevalence of dental caries among diabetics is higher than a study in southwest Cameroon at 19.5% (29), Sheshdeh in Iran at 43% (40), in Gujarat, India, at 73.3% (36), in Shandong, China, 67% (23). The possible explanation for this difference may be the difference in population characteristics, diet, duration of diabetes, and treatment (23).

The prevalence of dental caries among nondiabetics was comparable with the study conducted in Asmara, Eritrea, 67.9% (17), and Brazil 68.5% (41). Whereas this is less than a study conducted in Bahir Dar, Ethiopia, 75.0% (42), in Debre Tabor, Ethiopia, 78.2% (19), Egypt 86.63% (15), in San Luis Potosi, Mexico, 76.5% (43). The reason for this may be age group differences, and population study differences, such as those visiting only dental clinics may increase the prevalence of dental caries. But, this prevalence of dental caries among nondiabetics is greater than in Adama, Ethiopia, with 35.10% (18) Finote Selam, Ethiopia, with 48.50% (44), Aksum, Ethiopia, with 35.4% (31), Gondar, Ethiopia, with 23.64% (45), in Butajira, Ethiopia, with 60% (46), Shashamane, Ethiopia, with 64.6% (47), and Port Harcourt, Nigeria, with 35.1% (48).

This study identifies the female gender as an independent predictor of the prevalence of dental caries. The prevalence of dental caries was significantly higher among those who were females compared to those who were males. This finding is consistent with the previous studies (16, 24, 43, 48). This is contradicted by a study in Adama, Ethiopia, which say males are more at risk (18). The reason for higher prevalence might be the early eruption of permanent teeth in females (49). So, being exposed to influent factors for a long time can raise the decay prevalence of permanent teeth among females more than males. Another reason could be hormonal fluctuation during puberty and salivary flow rate difference between females and males (50). On the other hand, a study conducted in Butajira reported no significant difference between males and females in dental caries experience (46).

Additionally, this study stated that not cleaning teeth was associated with the prevalence of caries. Other authors have reported similar findings in Finote Selam, Dessie, Russia (13, 24, 44). This may be due to the fact that toothbrushing removes away food debris from the mouth.

Likewise, this study reveals that xerostomia (feeling of dry mouth during meals) was a significant factor associated with dental caries. This finding is in agreement with the findings reported in Uganda and Pakistan (16, 33). This may be due to decreased saliva flow rate, which is a natural defense of oral health.

Furthermore, those who had khat chewing habits were found significantly associated with tooth decay. The study in Butajira town and the West Wollega zone in Ethiopia supports this finding (46, 51). On the other hand, this finding is in contrast to a study reported in Jimma, Ethiopia (26). The possible explanation for this may be khat chewer's sugary food intake like soft drinks and sugar with khat, which could have enhanced the association.

Furthermore, this study finding reveals that respondents who had poor knowledge related to dental caries had a significant association with dental caries as compared to those who had good knowledge about dental caries, supported by a study done in Ethiopia (19).

Moreover, this study found that poor oral hygiene status was also significantly associated with the prevalence of dental caries. This finding is consistent with studies reported in India, Cameroon, Iran, Spain, and India (29, 40, 43). Poor oral hygiene status means the oral with debris, which may provide nutrients and time for the bacteria to produce acid and finally tooth decay.

Finally, those who take tea with sugar were significantly associated with the occurrence of dental caries. Similar findings were reported in recent studies done in Mekelle town, Ethiopia (25), and Debre Birhan town, Ethiopia (52). It is well-recognized that sugar plays an important role in caries development. A sugary diet allows the bacteria to attach easily to the dental surface. The bacteria then convert into acids that cause demineralization of the hard tissue of the teeth.

In this study, the prevalence of dental caries was assessed among three socioeconomic groups. Socioeconomic status comparison using chi-square shows no association between diabetic and nondiabetic groups with a chi-square *p*-value = 0.473 (Table 1). Similarly, the multiple logistic regression analysis does not provide statistically significant evidence that supports the risk of socioeconomic status on dental caries prevalence (Table 6).

This study has its strengths. The first strength was study participants selected a consecutive sampling method and this reduces the selection bias. The second was questionnaire was adapted from WHO oral health survey fifth edition with some modifications. In addition, these studies were conducted in outpatient department patients including dental clinics. Further, the study includes factors like socioeconomic status using wealth index, oral hygiene status using oral hygiene index-simplified, and body mass index which are keys for dental caries development. And these risk factors are properly measured using their indexes.

This study also has limitations that should be taken into account when interpreting results. The first limitation is related to its design. Cross-sectional study design measures cause and effect at the same time. Therefore, it cannot establish temporal associations. Hence it was impossible to know the true causal order of disease. Dental caries were detected using clinical diagnosis only. It was not supported with radiological examination due to a lack of the instrument. This might affect the actual prevalence of the problem. In addition, the amount and duration of intake of sweet food items and drinks were not assessed. The other limitation of the study was not considering the metabolically controlled level of diabetics due to the lack of Glycosylated hemoglobin (HbA1c) instruments. Further, we recommend that future studies use the pulpal involvement ulcer due to root fragments fistula and abscess (PUFA) index to determine clinical conditions resulting from untreated dental caries.

## Conclusion

The prevalence of caries was significantly higher among diabetics than nondiabetics. Oral health education with preventive measures such as improving teeth cleaning practice, reducing sugary foods and drinks intake, and improving oral hygiene practice of patients should be mainstreamed along with diabetic follow-up care.

## Data Availability

The raw data supporting the conclusions of this article will be made available by the authors, without undue reservation.
